# The *Bactrocera dorsalis *species complex: comparative cytogenetic analysis in support of Sterile Insect Technique applications

**DOI:** 10.1186/1471-2156-15-S2-S16

**Published:** 2014-12-01

**Authors:** Antonios A Augustinos, Elena Drosopoulou, Aggeliki Gariou-Papalexiou, Kostas Bourtzis, Penelope Mavragani-Tsipidou, Antigone Zacharopoulou

**Affiliations:** 1 Department of Biology, University of Patras, Greece; 2 Insect Pest Control Laboratory, Joint FAO/IAEA Programme of Nuclear Techniques in Food and Agriculture, Seibersdorf, Vienna, Austria; 3 Department of Environmental and Natural Resources Management, University of Patras, Agrinio, Greece; 4 Department of Genetics, Development and Molecular Biology, School of Biology, Faculty of Sciences, Aristotle University of Thessaloniki, Thessaloniki, Greece

## Abstract

**Background:**

The *Bactrocera dorsalis *species complex currently harbors approximately 90 different members. The species complex has undergone many revisions in the past decades, and there is still an ongoing debate about the species limits. The availability of a variety of tools and approaches, such as molecular-genomic and cytogenetic analyses, are expected to shed light on the rather complicated issues of species complexes and incipient speciation. The clarification of genetic relationships among the different members of this complex is a prerequisite for the rational application of sterile insect technique (SIT) approaches for population control.

**Results:**

Colonies established in the Insect Pest Control Laboratory (IPCL) (Seibersdorf, Vienna), representing five of the main economic important members of the *Bactrocera dorsalis *complex were cytologically characterized. The taxa under study were *B. dorsalis s.s., B. philippinensis, B. papayae, B. invadens *and *B. carambolae*. Mitotic and polytene chromosome analyses did not reveal any chromosomal characteristics that could be used to distinguish between the investigated members of the *B. dorsalis *complex. Therefore, their polytene chromosomes can be regarded as homosequential with the reference maps of *B. dorsalis s.s.. In situ *hybridization of six genes further supported the proposed homosequentiallity of the chromosomes of these specific members of the complex.

**Conclusions:**

The present analysis supports that the polytene chromosomes of the five taxa under study are homosequential. Therefore, the use of the available polytene chromosome maps for *B. dorsalis s.s*. as reference maps for all these five biological entities is proposed. Present data provide important insight in the genetic relationships among the different members of the *B. dorsalis *complex, and, along with other studies in the field, can facilitate SIT applications targeting this complex. Moreover, the availability of 'universal' reference polytene chromosome maps for members of the complex, along with the documented application of *in situ *hybridization, can facilitate ongoing and future genome projects in this complex.

## Background

The *Bactrocera dorsalis *complex species is a group of true fruit flies belonging to Tephritidae, with great economic importance. Following the most recent taxonomic revisions, this complex is currently harboring approximately 90 morphological similar taxa [1,2]. Among them, eight are considered as economic important pests [2], including among others *B. dorsalis s.s., B. philippinensis, B. papayae *and *B. carambolae*. In 2003, an addition to the complex was made: *B. invadens *was detected in Kenya, and initially was considered a morphological variant of *B. dorsalis s.s*. [3]. However, in the following years it was recognized as a different species within the *B. dorsalis *complex [4]. Ever since that revision in 2005, there were doubts regarding whether all these members really represent well-differentiated species, mainly due to the lack of robust diagnostic characters [5].

In recent years, accumulating data cast doubt on the 'actual' number of different species in the complex. Research performed by different laboratories points to a possible overestimation in the number of discrete taxa in the complex and the need of another taxonomic revision to incorporate the synonymic status of different species. This research includes morphological/morphometric studies [6-10], behavioral/sexual compatibility analysis [11,12], as well as chemoecological [13,13-15] and molecular genetic approaches [7-9,13,16-20]. Recently, Drew and Romig [1] have synonymized *B. papayae *with *B. philippinensis*; however there is also an ongoing debate about the species status of other important pests of the complex.

The delimitation of species within the *B. dorsalis *complex is not just a scientific question regarding evolution and speciation. It is also important for the agricultural economies of countries that heavily rely on fruit exports. The first aspect refers to quarantine measures. The current taxonomy leads to the implementation of certain quarantine policies; therefore it is critical to be as accurate as possible, when assessing species limits of economic important pest populations. As a characteristic example, the description of *B. invadens *as a separate species within the *B. dorsalis *complex prompted additional fruit export restrictions in many African countries, leading to increased economic losses [7,12]. The second aspect involves the effectiveness of SIT applications. SIT is probably the most environmental friendly pest control method since it is species specific and does not result in chemical or biological pollution. The main principle of SIT is the release of sterile flies in the field. Mating of sterile laboratory flies with the targeted population leads to infertile crosses and subsequent population suppression. Successful SIT is facilitated by a) the clarification of genetic relationships among targeted populations and laboratory strains and b) the availability of well characterized, stable and competitive genetic sexing strains (GSSs) that allow the release of only males into the field. The importance of stable and competing GSSs in SIT is well documented in the Tephritidae SIT model organism, *Ceratitis capitata *[21-23]. In principle, male only releases are more effective since they can lead to a) increased efficiency of sterile males in the field and b) better fruit quality, avoiding damage from released females. Today, there are only few GSSs for the *B. dorsalis *complex, initially developed for the control of *B. dorsalis s.s*. [24-26]. The creation of such strains through classical genetic approaches is species-specific and not an easy task. Thus, exploring the possibility of universal use of the same GSS for some of the economic important members of the complex could facilitate their control. The promising results of [27,28], showing the possibility of controlling *B. carambolae *with *B. dorsalis s.s*. sterile flies point in such a direction.

Species limits can sometimes be obscure, and speciation can be driven by a variety of forces. Among them, chromosomal rearrangements (mainly inversions), are considered as key factors in Diptera speciation, especially in sympatric populations [29]. Early cytogenetic studies in *Drosophila*, based on mitotic and polytene chromosomes, were the first to detect interspecific inversions' differences [30,31]. Sturtevant and Dobzhansky [32] and Dobzhansky [33] first showed that chromosome inversions can be used to study the evolutionary history of a species group. Within this frame, inversions were proposed to have an important role in genetic variation and speciation leading thus to their extensive use as interspecific phylogenetic markers. The recent accumulation of comparative genomic data from *Drosophila *species [34-38] and mosquitoes [39-41] supports the importance of inversions in the suppression of gene flow in hybridizing taxa. Many models had been proposed regarding how inversions can enforce or support speciation, focusing mainly in the fitness of heterokaryotes (for a review see [42]). More recent theories, supported by genomic data, point to the suppression of recombination within and near inversions as a mechanism leading to reduced gene flow and maintenance of genetic divergence [38,42,43]. A possible role of an inversion can be the 'protection' of a combination of locally co-adapted alleles from introgression [44], that can lead to further accumulation of differences and facilitate speciation.

Taking into account the above, it is evident that cytogenetic analyses can help in resolving species boundaries within species complexes. This has been well documented in different *Drosophila *species [45], such as the endemic Hawaiian picture-winged group [46] and the American *repleta *species group [47]. In respect to this, the availability of polytene chromosomes in different Tephritidae genera, like *Ceratitis *[48], *Bactrocera *[49-53], *Dacus *[54], *Rhagoletis *[55-57,57] and *Anastrepha *[58] is valuable when seeking characteristic and diagnostic differences in closely related species.

Studies in *B. dorsalis *complex have also demonstrated the importance of adequate and well characterized samples: when exploring species limits and characters that may overlap, it is important to develop well organized and comprehensive sampling schemes [8,16]. Since species limits can be fuzzy and different classes of markers can provide different levels of resolution, the use of all available tools for species identification is highly desirable.

In the present study, we tried to identify chromosomal differences between five of the main agricultural pests of the complex, namely *B. dorsalis s.s., B. philippinensis, B. papayae, B. invadens *and *B. carambolae*, through the analysis of their mitotic complements and the comparison of their polytene chromosomes with the published reference maps for *B. dorsalis s.s*. [50]. As working material, samples representing well characterized colonies of these species, held at the Insect Pest Control Laboratory (IPCL, Seibersdorf, Vienna), were used. These colonies have been used in a variety of FAO/IAEA projects [8,11-13,16], and their status has been verified repeatedly. Polytene chromosomes derived from two F_1 _bidirectional hybrids (*B. dorsalis s.s*. × *B. invadens *and *B. dorsalis s.s*. × *B. carambolae*) were also analyzed, aiming at the detection of fixed chromosomal rearrangements among the parental colonies. We focused on these hybrids since: a) *B. invadens *is the only member of the complex originating from Africa, and its current recognition as a distinct species within the complex has severe quarantine consequences and b) *B. carambolae *is considered to be more clearly differentiated from the other four members of the complex [10,11,16]. Finally, *in situ *hybridization was performed using unique genes, attempting to: a) provide diagnostic landmarks for the polytene chromosome arms, b) reveal small chromosome rearrangements undetectable by microscopic observation and c) test the utility of *B. dorsalis *complex polytene chromosomes and polytene maps for future mapping experiments.

## Methods

### B. dorsalis complex strains

Colonies representing the five economic important members of the complex currently established at the IPCL were used. Specifically, two colonies of *B. dorsalis s.s*. (Saraburi - Thailand and Nakhon Si Thammarat - Thailand), one of *B. philippinensis *(Philippines), one of *B. papayae *(Serdang-Malaysia), one of *B. invadens *(Kenya) and one of *B. carambolae *(Paramaribo, Suriname) were analyzed. In addition, the two following F_1 _bidirectional hybrids were analyzed: a) *B. dorsalis s.s*. (Saraburi strain) × *B. carambolae *and b) *B. dorsalis s.s*. (Saraburi strain) × *B. invadens*.

### Mitotic chromosome preparations

Chromosome preparations were made, as described in [48]. Brain tissue from third instar larvae was dissected in 0.7 % NaCl, transferred to 1 % sodium citrate on a well slide for at least 15 min and fixed in fresh fixation solution (methanol/acetic acid 3:1) for 3min (fixation solution was changed twice in this step). Fixation solution was removed and a drop of acetic acid (60 %) was added. Tissue was dispersed using a micropipette and the cell suspension was dried by laying it on a clean slide placed on a hotplate (40-45 ^o^C). Chromosomes were stained with Giemsa (5 % Giemsa in 10 mM phosphate buffer, pH 6.8). Chromosome slides were analyzed at 100 × magnification, using a phase contrast microscope (Leica DMR), and photographs were taken using a CCD camera (ProgRes CFcool; Jenoptik Jena Optical Systems, Jena, Germany). At least 15 good quality preparations (each one representing one larva) per sample and at least 10 well spread nuclei per preparation were analysed.

### Polytene chromosome preparations

Polytene chromosome preparations were made from 3^rd ^instar larvae, as described in [48]. Larvae were dissected in acetic acid (45 %), and salivary glands were transferred to HCl (3 N) for 1 min, fixed in 3:2:1 fixation solution (3 parts acetic acid: 2 parts water: 1 part lactic acid) for ~5 min (until transparent) and stained in lactoacetic orcein for 5-7 min. Glands were washed with 3:2:1 solution to remove excess stain and squashed. Chromosome slides were analyzed at 100 × magnification using a phase contrast microscope (Leica DMR) and photographs were taken using the ProgRes CFcool CCD camera. At least 25 good quality preparations (each one representing one larva) per sample and at least 10 well spread nuclei per preparation were analysed.

### In situ hybridization

Polytene chromosome preparations for *in situ *hybridization were made from salivary glands of 1-4 day-old pupae, as described in [59]. Six heterologous gene sequences originating from other tephritid species were used as probes (Table 1). Labeling and detection was performed using the DIG DNA Labeling and Detection kit (ROCHE Diagnostics, Mannheim, Germany), according to [60]. Hybridization was performed at 60 °C. Two to three preparations per strain were hybridized with each probe, and at least ten well spread nuclei per preparation were analyzed.

**Table 1:** The hybridization probes used in the present study and their localization sites on the polytene chromosomes of the five taxa studied from the *B. dorsalis* species complex.

Gene name	Description	Species of origin	DNA type	Reference	Localization site
***hsp70***	the *heat-shock 70 *gene	*Ceratitis capitata*	genomic	[74]	**26-3L**
***gld***	the *glutamate dehydrogenase *gene	*Ceratitis capitata*	genomic	unpublished	**6-2L**
***scarlet***	the orthologue of the *scarlet *gene of *D. melanogaster*	*Bactrocera tryoni*	genomic	[75]	**82-6L**
***ovo***	orthologue of the *ovo *gene of *D. melanogaster*	*Bactrocera oleae*	cDNA	unpublished	**63-5L**
***sxl***	orthologue of the *sex lethal *gene of *D. melanogaster*	*Bactrocera oleae*	cDNA	[76]	**78-5R**
***tra***	orthologue of the *transformer *gene of *D. melanogaster*	*Bactrocera oleae*	cDNA	[77]	**86-6L**

The localization site was determined according to the *B. dorsalis s.s*. polytene maps [50]

## Results

### Mitotic karyotype analysis

**Figure 1:**
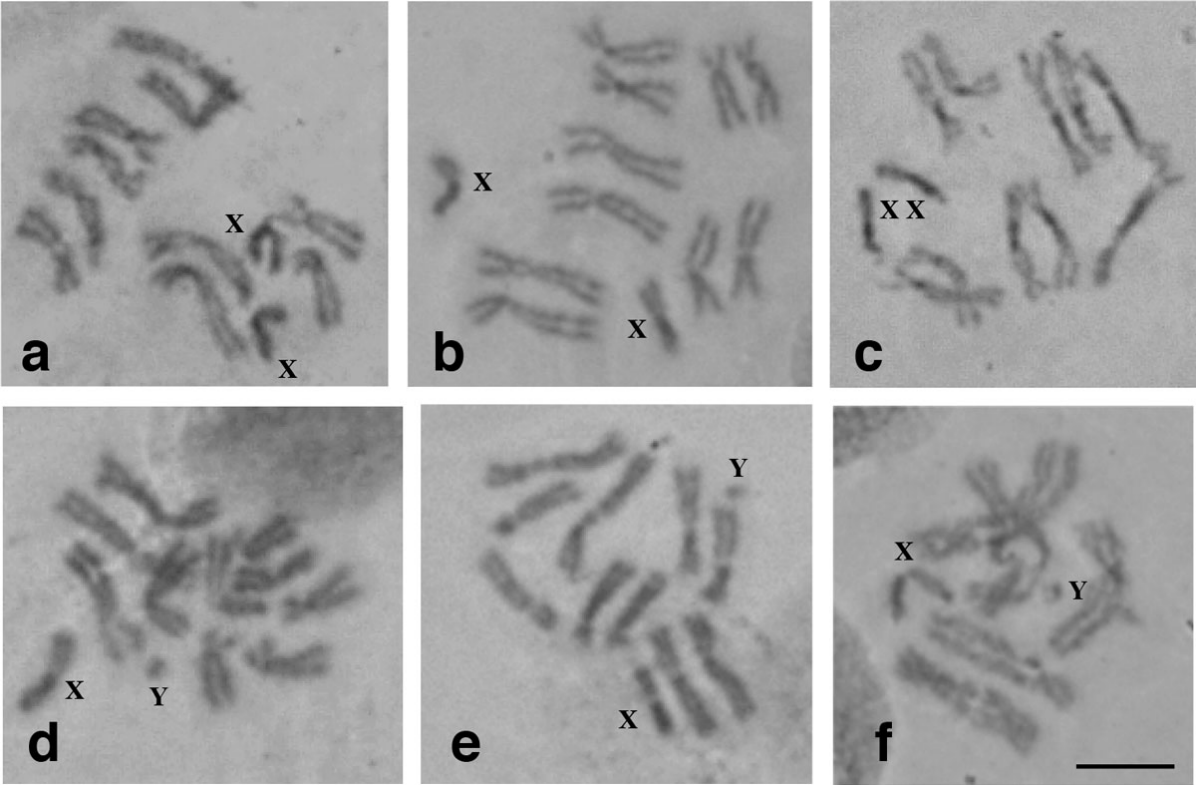
**Mitotic karyotypes of members of the *B***. *dorsalis *species complex. **a **and **d) ***B. dorsalis s.s*. (Saraburi), **b) ***B. papayae*, **c) ***B. invadens*, **e) ***B. carambolae*, **f) ***B. philippinensis*. **a-c) **females, **d-f) **males. Scale bar represents 5 μm.

All the members of the complex analyzed here (*B. dorsalis s.s., B. philippinensis, B. papayae, B. invadens *and *B. carambolae*) show five pairs of autosomes and one pair of heteromorphic sex chromosomes (XX/XY). The autosomes have been numbered II to VI according to descending size order [50]. The two longest (II and III) and the two shortest (V and VI) autosomes can be described as submetacentric, although with different arm ratios, and one autosome (IV) can be described as metacentric. The sex chromosomes are the smallest of the set, with the × being elongated, metacentric, with one of the arms being darker stained than the other and the Y being dot-like (Figure 1). The observed karyotype is referred as form A [61]. No differences in the karyotypes were observed.

### Polytene chromosome analysis

No evidence of polytenization of the sex chromosomes was observed. This in accordance with the polytene complement published for *B. dorsalis s.s*. [50].

**Figure 2:**
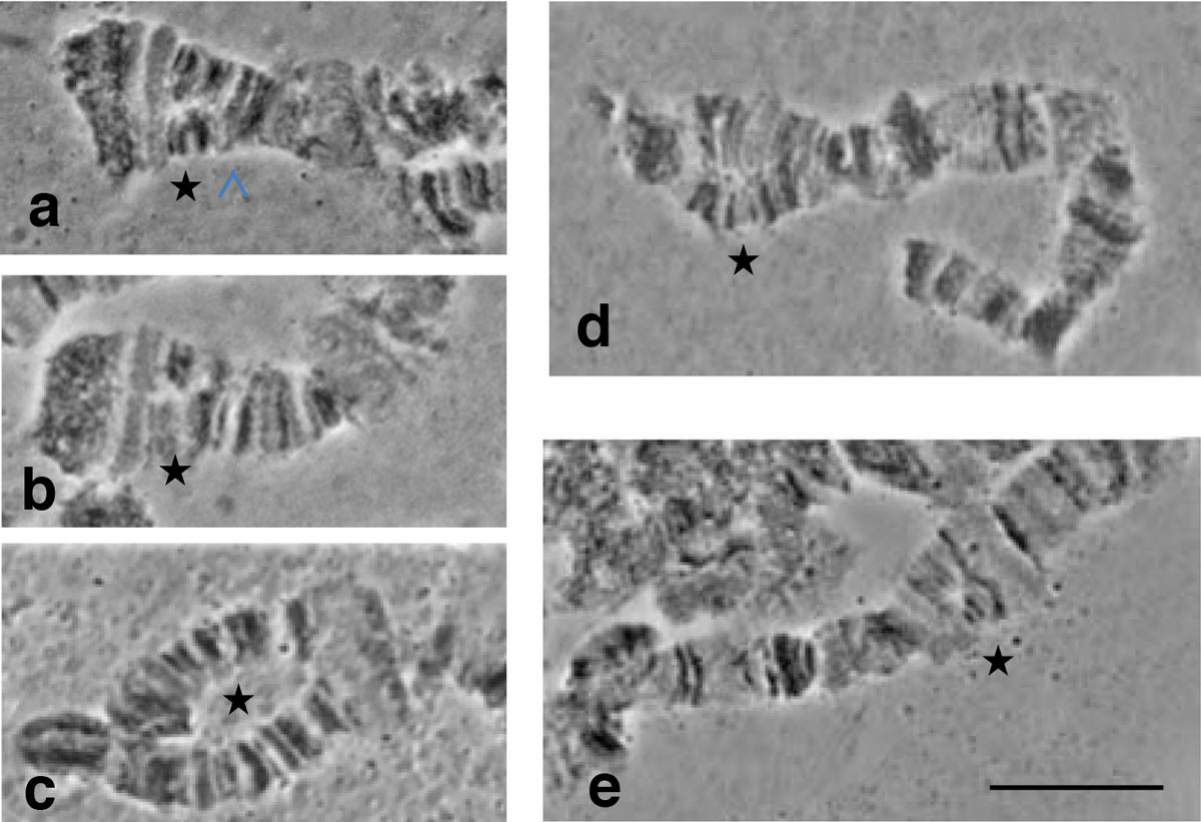
**Polymorphic asynapses in different polytene chromosome regions**. Variations in the appearance of the asynaptic region 73-74 of chromosome arm 5R: **a) **in *B. carambolae *and **(b, c) **in the *B. dorsalis s.s*. × *B. carambolae *hybrid. Minor asynapses in the *B. dorsalis s.s*. × *B. carambolae *hybrid within: **d) **regions 43 of chromosome arm 4L and **e) **regions 78-79 of chromosome arm 5R. Asterisks indicate the asynaptic regions. Scale bar represents 10 μm.

A comparison of the polytene elements of all analyzed strains with the reference map of *B. dorsalis s.s*. [50] revealed perfect correspondence of the banding patterns. No fixed chromosome rearrangements were detected. Consequently, all the strains can be regarded as homosequential and the available polytene chromosome maps of *B. dorsalis s.s*. can be used for all of them. Furthermore, the heterochromatic mass of the centromeric regions was identical in quality and quantity in all analyzed members of the complex, providing a useful landmark for the identification of each polytene chromosome. The characteristic polymorphic asynapsis on the right arm of chromosome 5 (sections 73-74), previously found in *B. dorsalis s.s*. [50], was also observed at varying frequencies (10-50 %) in all samples (Figure 2a-c). A few additional minor polymorphic asynapses were distributed over the polytene arms (Figure 2d-e).

**Figure 3:**
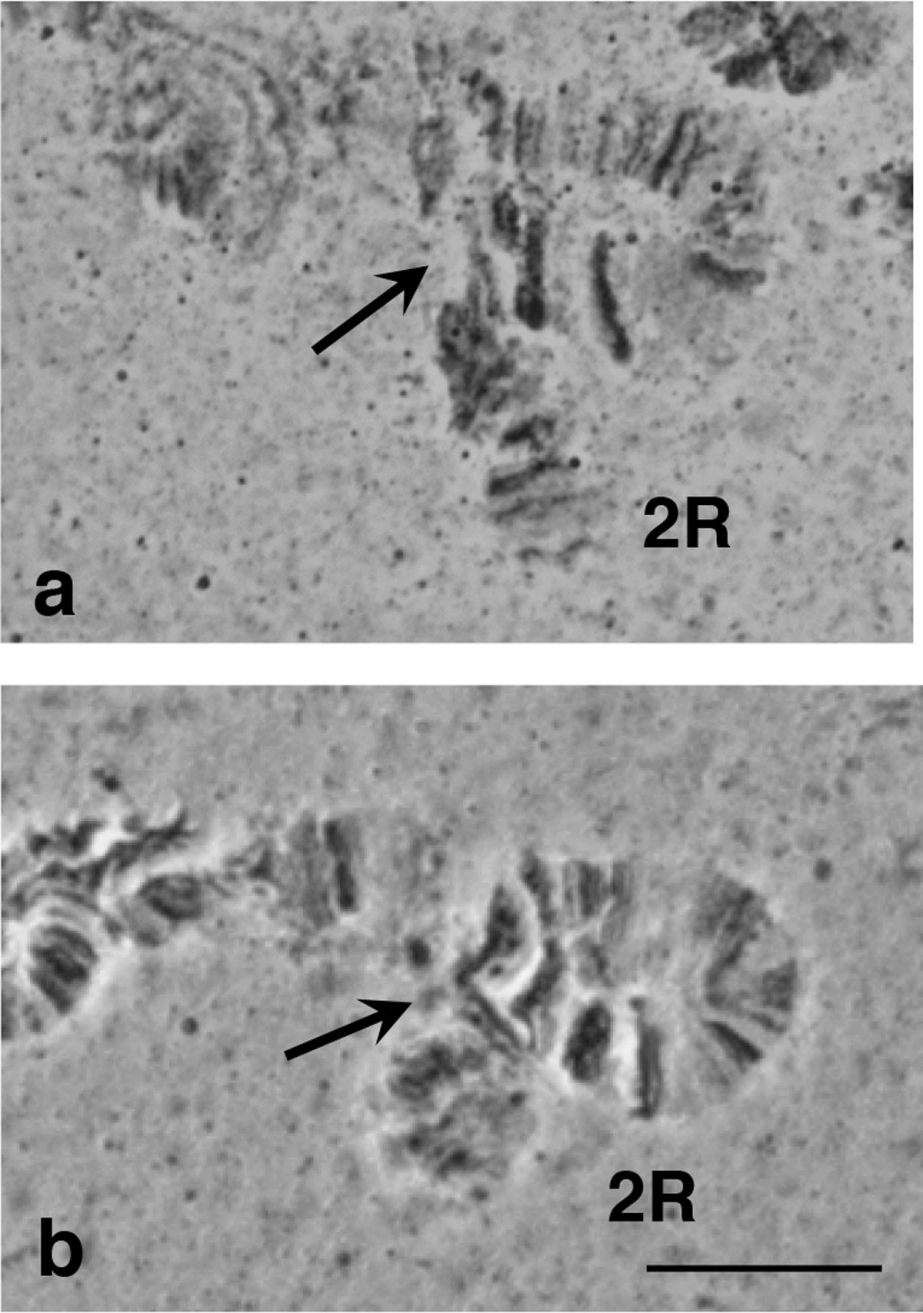
**The polymorphic inversion on the 2R polytene chromosome arm**. **a) **A polymorphic inversion found in the distal part of chromosome arm 2R in the Saraburi colony of *B. dorsalis s.s.*, **b) **the same inversion in the *B. dorsalis s.s*. × *B. invadens *hybrid. Arrows indicate the breakpoints. Scale bar represents 10 μm.

Polymorphic inversions were found on chromosome arm 2R of the two *B. dorsalis s.s*. samples (Figure 3). This is in accordance with the data of [50]. No other members of the complex showed any polymorphic inversions.

### Polytene chromosome analysis of F_1 _hybrids

**Figure 4:**
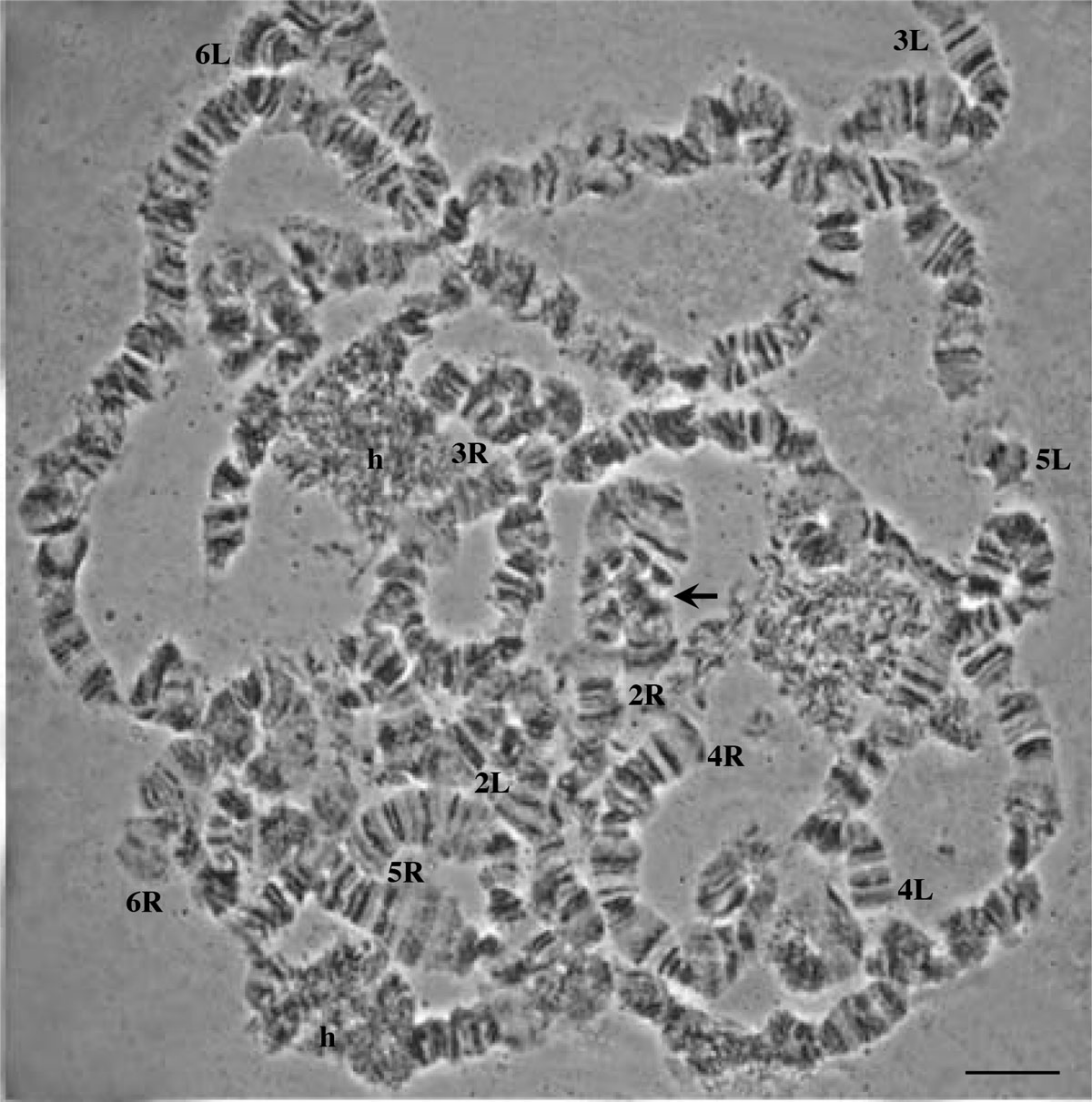
**Polytene nucleus of the F_1 _hybrid of *B. dorsalis s.s*. × *B. carambolae***. Note the perfect synapsis along the parental homologous chromosomes. Arrow indicates the polymorphic inversion found in the distal part of 2R chromosome arm in the Saraburi colony of *B. dorsalis s.s*. The chromosome tips are indicated; *h *indicates pericentric heterochromatin. Scale bar represents 10 μm.

**Figure 5:**
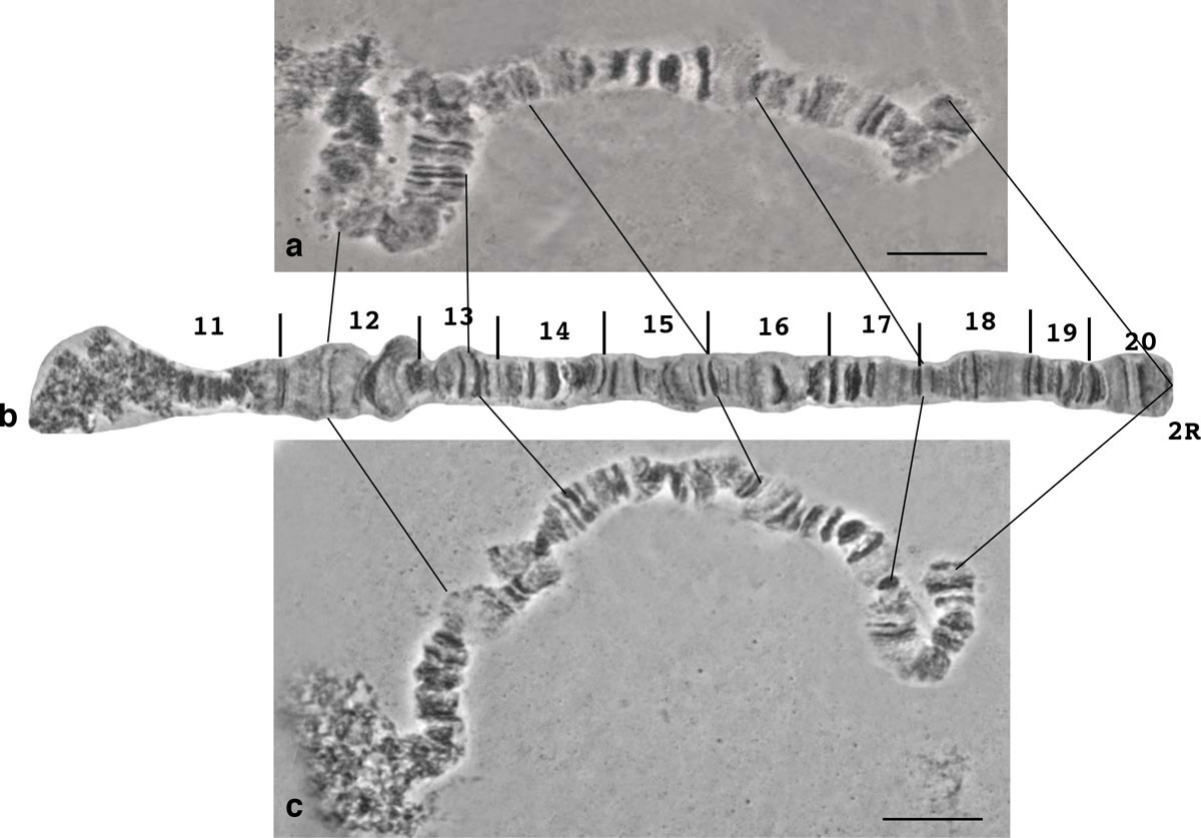
**Comparison of the 2R chromosome arm between *B. dorsalis s.s*. and its hybrids with *B. carambolae *and *B. invadens***. **a) **chromosome arm 2R of the F_1 _hybrid of *B. dorsalis s.s*. × *B. carambolae*, **b) **reference map of chromosome arm 2R of *B. dorsalis s.s*. and **c) **chromosome arm 2R of the F_1 _hybrid of *B. dorsalis s.s*. × *B. invadens*. Note the banding pattern similarity. Scale bar represents 10 μm.

In order to verify the identical banding pattern of the analyzed species, cytological analysis of F_1 _*B. dorsalis s.s*. × *B. invadens *(bidirectional) and F_1 _*B. dorsalis s.s*. × *B. carambolae *hybrids (bidirectional) was performed. The analysis of chromosome preparations of the hybrids did not reveal signs of fixed chromosome differences between the parental strains, evident from the perfect synapses among the parental homologous chromosomes (Figure 4). The comparison with the reference polytene chromosome maps of *B. dorsalis s.s*. verified that hybrids and their parental strains are homosequential with *B. dorsalis s.s*. (Figure 5). In both hybrids, similar to the parental strains, the asynapsis at region 73-74, together with some other minor polymorphic asynapses were observed (Figure 2b-e). The number of minor asynaptic sites was higher in the *B. dorsalis s.s. × B. carambolae *F_1 _hybrids than the *B. dorsalis s.s. × B. invadens *F_1 _hybrids.

### In situ localization of genes

**Figure 6:**
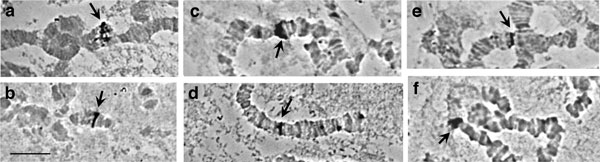
**Hybridization sites of six different probes on salivary gland polytene chromosomes of the *B. dorsalis *complex species. a) ***gld *in the *B. dorsalis s.s*. × *B. invadens hybrid*, **b) ***hsp 70 *in *B. dorsalis s.s*. × *B. carambolae *hybrid, **c) ***ovo *in *B. dorsalis s.s*. × *B. carambolae *hybrid, **d) ***sxl *in *B. dorsalis s.s*. × *B. carambolae *hybrid, **e) ***scarlet *in *B. dorsalis s.s*. and **f) ***tra *in *B. dorsalis s.s*. × *B. carambolae *hybrid. Arrows point to the hybridization signals. Note that signals in the hybrids show no differences between the two parental homologous chromosomes. Scale bar represents 10 μm.

*In situ *localization of six unique genes, namely *gld, hsp70, ovo, sxl, scarlet *and *tra *(Table 1) was performed on the polytene chromosomes of the five taxa, as well as on the two hybrids. Each probe yielded a unique signal at the same chromosomal position in all entities. More specifically, *gld *localized in region 6 of 2L, *hsp70 *in region 26 of 3L, *ovo *in region 63 of 5L, *Sxl *in region 78 of 5R and *scarlet *and *tra *in regions 82 and 83 of arm 6L (Table 1, Figure 6).

## Discussion

The main findings of the present study can be summarized as follows: a) mitotic karyotypes of the five members of the complex presented form A, the typical one for *B. dorsalis s.s*. which, according to [61] represents the most ancestral form of the complex, b) polytene chromosome analysis of both parental strains and selected F_1 _hybrids did not reveal any fixed differences among the five members of the complex, and c) *in situ *hybridization of selected genes confirmed that there are no differences among the five members of the complex based at least on the limited number of probes tested. The *in situ *results also provided characteristic landmarks for the recognition of the polytene arms and demonstrated the utility of polytene chromosomes and reference maps of the complex for *in situ *mapping projects.

### Implications for SIT applications

The *B. dorsalis *species complex includes at least eight economic important pests [2] that infest a variety of hosts worldwide and are putative targets for SIT. The development of GSSs, a prerequisite for efficient and cost-effective SIT programs, has been already achieved for *B. dorsalis s.s*. [24-26]. However, the availability of such strains does not mean that they are *a priori *suitable for mass rearing and release purposes. These strains have to exhibit a number of traits, such as genetic stability and good productivity in the laboratory or in mass rearing conditions over a number of generations as well as male mating competitiveness in the field. In respect to this, cytogenetic knowledge of the chromosomal events generating GSSs, along with standard Quality Control (QC) measures are of major importance for the assessment of the abovementioned traits [62].

The resolution of biological relationships among the different entities of species complexes is of high importance, since SIT application without this knowledge could jeopardize the effectiveness of such programs, especially in areas where different members of the complex overlap. This information is very useful in respect to the selection of appropriate laboratory strains for release purposes. The findings of the present study along with other studies that support a single species scenario [7-9,11,13,16,17] at least for the four of the five economic important members of the complex studied (*B. dorsalis s.s., B. papayae, B. philippinensis *and *B. invadens*), favour the 'universal' application of the *B. dorsalis s.s*. GSSs against all the above members of the complex. This is very important, considering the effort required in generating GSSs through classical genetic methods. In respect to this, the recent study of [27] presented in the same special issue points to the possibility of using the *B. dorsalis s.s*. GSS against *B. carambolae*, after several generations of crosses aiming to integrate this strain to *B. carambolae *genomic background.

### Mitotic karyotypes - no evidence of differentiation

Our analysis of mitotic chromosomes shows that all members of the complex studied here exhibit the same karyotype, described previously as form A [61]. This form is assumed to be the ancestral form in the complex and typical of *B. dorsalis s.s*.[50,61,63-65]. However, Baimai et al. [63] had previously described a different mitotic karyotype for *B. carambolae*. In that study, samples derived directly from infested fruits and characterized as *B. carambolae *based on morphological, geographic and host criteria, were reported to possess × chromosomes larger than the autosomes (form E karyotype). Our analysis does not confirm this report. A recent cytogenetic study on a *B. carambolae *colony derived from Malaysia also presented a typical form A karyotype for this species [66].

Given that a) geographic origin and plant host alone cannot be regarded as absolute taxonomic criteria [9,10,19] and b) it is difficult to establish robust morphological diagnostic characters for the different members of the complex [7,9,13,16,17,19], it is apparent that one must be quite skeptical regarding accurate species identification based only on these parameters. To avoid such problems, in the present study we used only material from IPCL. This is colonized material and therefore available at any time for different types of analysis.

Previous studies on mitotic karyotypes of the *B. dorsalis *complex have shown that there is considerable variability in size and ratio of the X chromosome arms [61,63-65]. X and Y size polymorphism has also been observed in other tephritid species complexes, including the *A. fraterculus *complex [67]. Τhe highly heterochromatic nature of the sex chromosomes in all tephritids analyzed so far (evident also from the lack of polytenization due to their under-replication) [48-51,53-58,68,69] points to a possible increased 'tolerance' in gain and loss of material in these chromosomes, which could explain its size plasticity.

### Polytene chromosome analysis - no evidence of speciation mediated by chromosomal rearrangements

The proposed chromosomal homosequentiality of the five members of the *B. dorsalis *species complex is based on the following observations: i) absence of fixed chromosomal rearrangements in comparison to the reference map of *B. dorsalis s.s*.; ii) absence of differences among the parental homologous chromosomes in the two hybrids studied; iii) identical heterochromatic mass of the centromeric regions of each chromosome element in all taxa; iv) common characteristic asynapsis of the chromosomal region 73-74 and v) *in situ *localization of each of six genes on the same chromosomal region in all taxa analyzed.

**Figure 7:**
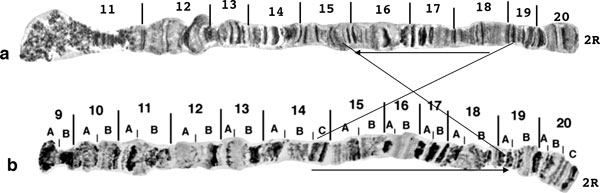
**Comparison of chromosome arm 2R between *B. dorsalis s.s.* and *B. tryoni*. a) **Chromosome arm 2R of *B. dorsalis s.s*. and **b) **chromosome arm 2R of *B. tryoni*. Note the fixed inversion between the two species.

In tephritid flies, genomic data are still scarce, and polytene chromosome maps are restricted to a few species. However, comparative polytene chromosome analysis and *in situ *mapping of unique genes show that chromosomal rearrangements characterize different species [50,51,53,58,69], suggesting their possible involvement in speciation. A comparative analysis of polytene chromosome maps of *B. dorsalis s.s*. and *B. tryoni*, a species outside, but closely related to the *B. dorsalis *complex, clearly shows at least one fixed inversion in polytene arm 2R that differentiates the two species (Figure 7).

Even though fixed rearrangements were not found in the polytene chromosome of the species studied, polymorphic inversions were observed in the two *B. dorsalis s.s*. populations, as well as in the two hybrids (derived from the *B. dorsalis s.s*. genome). A similar observation has been reported for another Thailand *B. dorsalis s.s*. population [50]. Although not reported in other tephritids, polymorphic inversions are common in Diptera and their presence and frequencies usually differ between geographical populations within the species [31]. The cytogenetic analysis of more species of the *B. dorsalis *complex could provide important insight in the involvement of chromosomal rearrangements in speciation within this species group.

The minor polymorphic asynapses observed in all samples most probably represent differential gene expression of the two parental homologous chromosomes. However, the presence of small, undetectable (with microscopic observation) rearrangements, such as inversions, deletions or insertions of repetitive or heterochromatic material, cannot be excluded. Indeed, even small inversions can alter the control of regulatory elements and lead to differential gene expression (puffing activity) [43]. Thus, the higher number and frequency of small polymorphic asynapses observed in the *B. dorsalis s.s*. × *B. carambolae *hybrid, in respect to the *B. dorsalis s.s*. × *B. invadens *hybrid, may indicate that the *B. carambolae *genome has small differences compared to the other members of the complex. Current literature tends to support *B. carambolae *as a discrete entity within the complex, but closely related to the others [10,13,16,19]. The ability of *B. carambolae *to a) mate, although with reduced compatibility, b) produce viable and fertile progeny in the lab and c) produce hybrids carrying intermediate characteristics with other members of the complex [11,28,70,71] points to the presence of mainly prezygotic isolation between *B. carambolae *and the other members of the complex. Therefore, this is a case most likely representing incipient rather than complete speciation, a phenomenon also observed in the *A. fraterculus *complex [67,69].

## Conclusions

The present study sheds important light in the delimitation of species boundaries within the *B. dorsalis *species complex. Our data are in accordance with other recent studies questioning the currently accepted number of discrete species within this complex, since no fixed chromosomal differences were found. This outcome is of major importance for SIT applications targeting the different members of the complex. Currently, there are efforts towards genome/transcriptome sequencing of the *B. dorsalis *complex [72,73] that are generating a great amount of sequences with, however, limited information regarding their overall organization. The comparative cytogenetic analysis presented here, accompanied with the *in situ *hybridization of genes on the polytene chromosomes, highlight the importance of cytogenetics in gaining more insight regarding organization of newly generated contig sequences and chromosomal localization of genes of specific interest.

## Competing interests

No competing interests exist.

## Authors' contributions

AAA, AGP, AZ conceived the experiments. AAA, ED, AGP, PMT, AZ performed the experiments. AAA, ED, AGP, KB, PMT, AZ performed the analysis. AAA, ED, AGP, KB, PMT, AZ wrote the manuscript. All authors read and approved the manuscript.
